# A review of combined sewer overflows as a source of wastewater-derived emerging contaminants in the environment and their management

**DOI:** 10.1007/s11356-021-14103-1

**Published:** 2021-04-29

**Authors:** Bruce Petrie

**Affiliations:** School of Pharmacy and Life Sciences, Robert Gordon University, Aberdeen, AB10 7GJ UK

**Keywords:** Wastewater, Emerging contaminant, Micropollutant, Pharmaceutical, Combined sewer overflow, Chiral

## Abstract

Emerging contaminants such as pharmaceuticals, illicit drugs and personal care products can be released to the environment in untreated wastewater/stormwater mixtures following storm events. The frequency and intensity of combined sewer overflows (CSOs) has increased in some areas due to increasing urbanisation and climate change. Therefore, this review provides an up-to-date overview on CSOs as an environmental source of emerging contaminants. Other than compounds with high removal, those chiral species subject to enantioselective changes (i.e. degradation or inversion) during wastewater treatment can be effective markers of CSO discharge in the environment. A proposed framework for the selection of emerging contaminants as markers of CSOs is outlined. Studies have demonstrated that CSOs can be the main source of emerging contaminants with high removal efficiency during wastewater treatment (e.g. > 90%). However, the impact of CSOs on the environment is location specific and requires decision-making on their appropriate management at catchment level. This process would be aided by further studies on CSOs which incorporate the monitoring of emerging contaminants and their effects in the environment with those more routinely monitored pollutants (e.g. pathogens and priority substances). Mitigation and treatment strategies for emerging contaminants in CSOs are also discussed.

## Introduction

Wastewater-derived emerging contaminants such as over-the-counter and prescription pharmaceuticals, illicit drugs, personal care product ingredients and food-related compounds are ubiquitous in surface waters globally (Ellis 2006; Zuccato et al. 2008; Lange et al. 2012; Hughes et al. 2013). More than 200 of these compounds have been found in the environment with concentrations typically in the ng L^−1^ to μg L^−1^ range (Hughes et al. 2013). In recent years, emerging contaminants have been subject to extensive research due to their potential threat to the ecology of receiving environments at these low concentrations (Petrie et al. 2015). For example, exposure of *Pimephales promelas* with the synthetic estrogen 17α-ethinylestradiol at 5–6 ng L^−1^ led to the collapse of a fish population in a Canadian Lake due to the feminisation of male fish (Kidd et al. 2007). The antidepressant oxazepam has been found to alter behaviour and feeding rate of *Perca fluviatilis* at 1.8 μg L^−1^ exposure concentrations (Brodin et al. 2013). Furthermore, the presence of antibacterial drugs and their potential to select for resistant bacteria is an emerging concern (Rizzo et al. 2013). An additional concern is the presence of emerging contaminants as complex mixtures in the environment which could result in synergistic effects (Schnell et al. 2009). In laboratory studies, considerable toxicity was observed for a mixture of nonsteroidal anti-inflammatory drugs (NSAIDs) at the same concentration where little effect was observed for the compounds individually (Cleuvers 2004).

Currently, in Europe, there is no legislation which governs the concentration of emerging contaminants in the environment. However, several were placed on a ‘watch list’ due to their suspected risk until further evidence is gathered (European Commission 2012; Carvalho et al. 2015). This included the steroid estrogens estrone, 17β-estradiol and 17α-ethinylestradiol, the NSAID diclofenac, and the macrolide antibiotics erythromycin, clarithromycin and azithromycin. Recent proposals have recommended the inclusion of further antibiotics, antifungals, steroids and antidepressants (Cortes et al. 2020). Determining environmental risk and development of legislation requires robust exposure and biological effect data sets. Monitoring the sources of these emerging contaminants is an essential step of the risk assessment process. This is also necessary for the development of appropriate control measures to lower their discharge. The main route of entry of these emerging contaminants to the environment is considered the release of treated effluents from municipal wastewater treatment plants (WTPs) (Gros et al. 2007; Kostich et al. 2014; Li 2014). Incomplete removal of emerging contaminants is observed during treatment by conventional WTPs as they were not designed for this purpose. A further notable source of emerging contaminants which has received comparatively less attention is combined sewer overflows (CSOs) (Phillips et al. 2012; Munro et al. 2019; Botturi et al. 2020; Brunsch et al. 2020; Mutzner et al. 2020). In such systems, untreated wastewater can be released directly to the environment during periods of heavy rainfall.

Therefore, the purpose of this review is to detail the progress made on understanding the role CSOs play in the dissemination of emerging contaminants in the environment. Emerging contaminant markers of untreated wastewater discharges, the effect of CSOs to environmental concentrations of emerging contaminants as well as mitigation and treatment strategies are discussed.

## Combined sewer overflows as a source of emerging contaminants

**Fig. 1:**
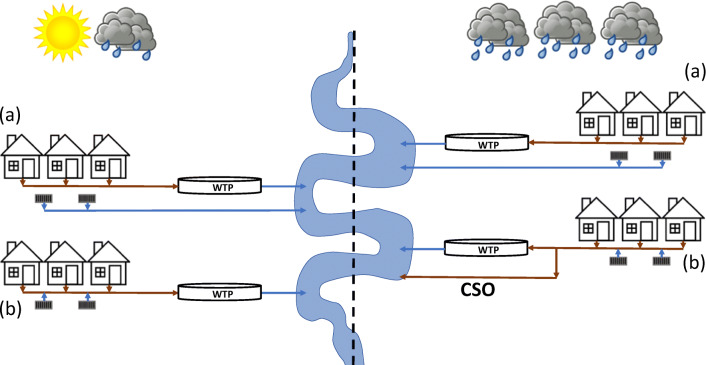
Separate sewer systems (a) and combined sewer systems (b) under ‘dry’ weather (left) and wet weather conditions (right). Key: WTP, wastewater treatment plant; CSO, combined sewer overflow

Generally, there are two different type of sewer systems used in Europe. A separate sewer system transports wastewater and surface run off separately (Fig. 1). Municipal and industrial wastewater is transported to a WTP for treatment whereas storm water is transported in a separate pipeline and discharged into a nearby watercourse, normally following physical treatment only (i.e., decantation). In a combined sewer, a single pipeline transports wastewater and surface runoff to a WTP (Fig. 1). Combined sewers often have capacity to deal with flows several fold above average ‘dry weather’ volumes experienced (Munro et al. 2019). However, capacity can be exceeded during periods of heavy rainfall or snowmelt. In such instances, a relief mechanism incorporated into the sewer system known as a CSO allows excess flows to be directed to a nearby watercourse without treatment (Botturi et al. 2020) (Fig. 1). Such discharges are essential to avoiding flooding of households and streets. Furthermore, exceeding the flow capacity of WTPs can result in damage to pipework as well as mechanical and electrical components. It should also be noted that many sewer systems are historical. Therefore, they are under added pressure from increased precipitation associated with climate change, paving of urban areas causing increased stormwater runoff, and new housing developments utilising the same sewer network (Schertzinger et al. 2019). Such factors increase the frequency and intensity of CSO discharges (Abdellatif et al. 2015).

In the UK, there are > 20,000 active consented discharges from storm overflows or pumping stations in England (Environment Agency 2020), and Scotland have approximately 4000 CSOs (Scottish Water 2020). To illustrate the frequency and extent of CSO discharges, there were on average 50-60 CSO discharge events per year (up to 2015) on the River Thames, UK, which resulted in the annual release of 39 million tonnes of untreated wastewater and stormwater (DEFRA 2015). Duration of CSO events can vary greatly. A study of 95 events in Switzerland found they can range from a few minutes to 96 h which was caused by snowmelt (median = 43 min) (Mutzner et al. 2020). As CSO discharges contain a mixture of untreated wastewater and stormwater, it may be expected that emerging contaminant concentrations are lower than those in untreated wastewaters during ‘dry weather’ conditions. The content of stormwater in CSOs has been calculated to range from 69 to 95% in studies conducted in France and Germany (Gasperi et al. 2012; Launay et al. 2016).

Madoux-Humery et al. (2013) found that median concentrations of caffeine, carbamazepine, paracetamol and theophylline in two CSOs were anywhere from 1.2 to 51.4 times lower than dry weather wastewater. On the other hand, Del Río et al. (2013) found that during rain events, the concentration of emerging contaminants can increase in combined sewers. Mean concentrations of carbamazepine, ibuprofen and paracetamol in combined sewer wastewater were 1.3 to 7.9 times greater during wet weather flows than under dry weather conditions (Del Río et al. 2013). It was postulated that this was due to their mobilisation through washout of particulate bound drug from sediments and biofilms in the sewer system. In a WTP in Stuttgart, Germany, it has been estimated that 10 to 65% of carbamazepine emissions during CSO events are attributed to this (Launay et al. 2016). Desorption of particulate bound pharmaceuticals has been demonstrated in laboratory studies simulating the addition of stormwaters to wastewaters (Hajj-Mohamad et al. 2017). Increased aqueous phase concentrations were observed despite dilution with stormwater.

In general, there is a lack of data for emerging contaminants in CSO discharges (Table 1). The interpretation of such data needs care due to the dynamic nature of CSO discharges and the number of factors (e.g., level of dilution) which influence emerging contaminant concentrations. At a WTP in Stuttgart, Germany, the variability of the mean emerging contaminant concentration across seven CSO events was about one order of magnitude (Launay et al. 2016). Nevertheless, the data reveals that CSO discharges are a source of emerging contaminants and they can be present at notable concentrations (e.g., > 1000 ng L^−1^) (Table 1). Kay et al. (2017) reported the concentrations of five emerging contaminants (diclofenac, erythromycin, ibuprofen, mefenamic acid and propranolol) in 14 different CSO samples collected in Northern England, UK. The concentrations reported were similar to those of WTP effluents and receiving waters except for the NSAID ibuprofen. Ibuprofen concentrations in CSO samples were an order of magnitude greater than WTP effluents, with a maximum concentration of 14,231 ng L^−1^ observed (Kay et al. 2017). A higher ibuprofen concentration in CSO discharges compared to treated effluents is likely to be a result of ibuprofen’s high removal efficiency (≥ 90%) during wastewater treatment (Buser et al. 1999; Petrie et al. 2016; Archer et al. 2017). Similarly, other emerging contaminants with high WTP removal efficiencies including steroid estrogens were found at concentrations in CSOs up to 10 times greater than treated effluents (Phillips et al. 2012).

**Table 1:** Concentration of emerging contaminants in combined sewer overflows

Emerging contaminant	Family/use	Monitoring strategy	Mean concentration (ng L^−1^)	Ref.
Diclofenac	NSAID	Grab samples of five CSOs collected during intensive rainfall. Number of replicates range from one to seven (14 samples in total). Samples collected from Aire and Calder catchments, Yorkshire, UK	74–388	A
Erythromycin	Antibiotic		< 5–1603	
Ibuprofen	NSAID		76–2734	
Mefenamic acid	NSAID		< 5–19	
Propranolol	Beta-blocker		< 5–11	
Ibuprofen	NSAID	Single grab sample of sewage overflow. Sample collected from Cooks River catchment, Sydney, Australia.	244	B
Naproxen	NSAID		25	
Caffeine	Stimulant	Grab samples collected by automated sampler during CSO events at two locations. Eight and two CSO events captured (125 and 10 samples collected, respectively). Samples collected from sewer of Greater Montreal Area, Canada.	270 and 3248	C
Carbamazepine	Antiepilepsy		184 and 4	
Paracetamol	Analgesic		3591	
Theophylline	Stimulant		2381 and 57	
Acesulfame	Sweetener	Volume-proportional samples collected during CSO events at one location (seven CSO events captured – 25 samples collected in total). Samples collected from WTP South-West of Stuttgart, Germany.	2965	D
Atenolol	Beta-blocker		41	
Bezafibrate	Lipid-regulator		90	
Caffeine	Stimulant		9030	
Carbamazepine	Antiepilepsy		84	
Diatrizoate	Contrast agent		19	
Diclofenac	NSAID		157	
Galaxolide	Musk		184	
Ibuprofen	NSAID		1239	
Iohexol	Contrast agent		144	
Iomeprol	Contrast agent		207	
Iopamidol	Contrast agent		95	
Iopromide	Contrast agent		212	
Metoprolol	Beta-blocker		200	
Naproxen	NSAID		118	
Propranolol	Beta-blocker		9	
Sulfamethoxazole	Antibiotic		23	
Sucralose	Sweetener		752	
Tonalide	Musk		31	
Triclosan	Antibacterial		122	
Caffeine	Stimulant	Grab samples of two CSOs following 24.5 mm of rainfall over 11 hours following six days without rainfall. Samples collected from Jung-rang Creek area, South Korea.	2149	E
Iohexol	Contrast agent		1165	
Iopamidol	Contrast agent		2394	
Iopromide	Contrast agent		940	
Carbamazepine	Antiepilepsy	Passive samplers deployed at three locations in Switzerland (10 events captured).	49–170^a^	F
Clarithromycin	Antibiotic		54–55^a^	
Diclofenac	NSAID		13–860^a^	
Carbamazepine	Antiepilepsy	Passive samplers deployed at 20 locations in Switzerland (95 events captured).	250–4800	G
Diclofenac	NSAID		78–1000	

A, Kay et al. 2017; B, Khan et al. 2014; C, Madoux-Humery et al. 2013; D, Launay et al. 2016; E, Ryu et al. 2014; F, Mutzner et al. 2019; G, Mutzner et al. 2020 Key: CSO, combined sewer overflows; NA, not applicable; NSAID, nonsteroidal anti-inflammatory; -, not measured

^a^Range presented

Other than reporting concentrations in CSOs, only a few studies have measured the released load (e.g. g day^−1^) of emerging contaminants in CSOs compared to treated effluents. Measuring load is a useful way of quantifying the contribution of CSOs as a source of emerging contaminants. An excellent study by Phillips et al. (2012) at a WTP in Vermont, USA, sampled influent and effluent wastewater as well as CSO discharges over a 13-month period. During this period, CSOs represented 10% of wastewater discharges. However, they accounted for 40–90% of released emerging contaminant loads with > 90% WTP removal (Phillips et al. 2012) (Table 2). Weyrauch et al. (2010) estimated that annual loads of compounds in the River Spree, Germany, with WTP removal efficiencies > 95% were predominantly from CSO discharges over treated effluent discharges. A study in the Maozhou River watershed, China reported that CSOs account for 97% of parabens discharged into the environment during rainfall events (Zhao et al. 2021).

**Table 2:** Multicompound studies aimed at investigating the effect of combined sewer overflows on emerging contaminants in the environment

Location	Sampling strategy			Target emerging contaminants for quantitative analysis	Analysis method		Findings	Ref.
	Mode	Sites	Frequency		Separation	Detection		
River Thames estuary, UK.	Grab	2	Daily on weekdays over 6-weeks. Composite influent and effluent wastewater samples also sampled to identify potential CSO markers.	Amitriptyline, antipyrine, bezafibrate, benzoylecgonine, caffeine, carbamazepine, chloramphenicol, clofibric acid, cocaine, dextromethorphan, diazepam, diclofenac, fluoxetine, furosemide, ketamine, ketoprofen, MDMA, mephedrone, metoprolol, nifedipine, nimesulide, nortriptyline, propranolol, sulfamethoxazole, sulfamethazine, sulfaphenazole, sulfapyridine, temazepam, tramadol, trimethoprim, warfarin.	UPLC C18	Orbitrap HRMS	Short-term increase of caffeine, cocaine and benzoylecgonine concentration (within an order of magnitude) following CSO events.	A
Aire and Calder catchments, UK.	Grab	7	Monthly over 18-months. Included sampling of wastewater effluents and two CSO discharges.	Diclofenac, erythromycin, ibuprofen, mefenamic acid, propranolol.	HPLC C18	Q-TOF MS/MS	Variability in concentrations observed but no correlation made to rainfall or CSO events.	B
Körsch catchment, Germany.	Grab	5	Nine samples collected during dry weather and following four CSO discharges. Influent wastewater and CSO discharges also sampled using composite samplers.	Acesulfame, atenolol, bezafibrate, caffeine, carbamazepine, diatrizoate, diclofenac, galaxolide, ibuprofen, iohexol, iomeprol, iopamidol, iopromide, metoprolol, naproxen, propranolol, sulfamethoxazole, sucralose, tonalide, triclosan.	HPLC	LIT MS/MS	Diclofenac exceeded its AA-EQS (100 ng L^−1^) downstream of the CSO discharge (but upstream of the WTP effluent discharge) during wet weather in 25% of samples. The AA-EQS was not exceeded during dry weather. Diclofenac concentrations downstream of the CSO and WTP effluent discharges were lower during wet weather. However, all samples here exceeded the AA-EQS.	C
Jung-rang creek, South Korea.	Grab	5	Once during dry weather and wet weather conditions. Wet weather samples collected following 24.5 mm of rainfall during 11 h after 6 days without rainfall.	Acesulfame, atenolol, benzophenone, caffeine, carbamazepine, diclofenac, diltiazem, diphenhydramine, estrone, ibuprofen, iohexol, iopamidol, iopromide, gemfibrozil, meprobamate, naproxen, primidone, propylparaben, sucralose, sulfamethoxazole, triclocarban, triclosan.	UPLC C18	QQQ	34%^a^ lower cumulative concentration under wet weather conditions. However, individual compound concentrations not reported.	D
WTP, Burlington, US.	24h flow-weighted composites	3	Influent (*n* = 18) and effluent (*n* = 22), and CSOs (*n* = 10) sampled over 13 months.	3β-coprostanol, 11-ketotestosterone, 17β-estradiol, β-sitosterol, androstenedione, benzophenone, bisphenol-A, caffeine, cholesterol, *cis*-androsterone, dihydrotestosterone, epi-testosterone, estriol, estrone, galaxolide, testosterone, tri(2-butoxyethyl)phosphate, triclosan.	GC low polarity proprietary phase	QQQ	CSO represent 10% of wastewater discharges but account for 40-90% of released loads of emerging contaminant with > 90% WTP removal.	E
Jamaica Bay, US.	Grab	24	Maximum of three times during dry weather conditions. Seven further samples collected from various locations following storm.	Antipyrine, caffeine, carbamazepine, cimetidine, codeine, cotinine, diltiazem, fenofibrate, fluoxetine, hydrocodone, ketoprofen, metformin, nicotine, nifedipine, paracetamol, paraxanthine, ranitidine, salbutamol, sulfamethoxazole, trimethoprim, warfarin.	HPLC C18	Q-TOF MS/MS	Following the storm event, concentrations of nicotine and paracetamol were similar or greater than dry weather concentrations.	F

A, Munro et al. 2019; B, Kay et al. 2017; C, Launay et al. 2016; D, Ryu et al. 2014; E, Phillips et al. 2012; F, Benotti and Brownawell 2007

Key: CSO, combined sewer overflows; HPLC, high performance liquid chromatography; HRMS, LIT MS/MS, linear ion trap mass spectrometer; high-resolution mass spectrometry; MDMA, 3,4-methylenedioxymethamphetamine; QQQ, triple-quadrupole mass spectrometer; Q-TOF MS/MS, quadrupole-time of flight mass spectrometer; UPLC, ultra-performance liquid chromatography

^a^Cummulative concentration includes several pesticides and flame retardant

A challenge of monitoring emerging contaminants in CSOs is the accessibility of suitable sampling locations and the intermittent nature of CSOs. Studies have adopted grab sampling (Khan et al. 2014; Ryu et al. 2014; Madoux-Humery et al. 2013; Kay et al. 2017) or composite sampling in a volume (Launay et al. 2016) or flow-proportional manner (Phillips et al. 2012) to measure emerging contaminants in CSOs. Automated samplers are advantageous in that they can be triggered to collect grab or composite samples during storm events. Flow proportional composite sampling is recommended for monitoring wastewater streams which are dynamic in nature to obtain representative information (Ort et al. 2010). However, an alternative approach has proposed the use of passive samplers deployed in the sewer overflow system. They can be deployed in the CSO pipeline and become submerged during a storm event (< 36 h). Once collected, they can be used to estimate time-weighted average concentrations of emerging contaminants (Mutzner et al. 2019). Passive samplers are often not preferred for quantitative determinations in such instances due to uncertainties in determined analyte concentrations, particularly under variable flow conditions. Mutzner et al. (2020) used passive samplers to monitor emerging contaminants at 20 CSOs in Switzerland. At 19 CSOs, the concentration of diclofenac exceeded its (chronic) environmental quality standard (EQS). In several sites, the EQS was exceeded by more than an order of magnitude and would rely on dilution within the environment to not exceed the EQS (Mutzner et al. 2020). Chèvre et al. (2013) also noted that the contribution of CSOs alone can result in predicted-no-effect-concentrations of some compounds to be exceeded in the environment.

## Emerging contaminant markers of CSO discharge

CSO discharges in the environment can be identified by measuring markers of untreated wastewater. Studies have reported several emerging contaminants including stimulants, analgesics, NSAIDs and beta-blockers that can be used for this purpose (Table 3). Typically, these are compounds with considerable differences in concentration between treated and untreated wastewater. Therefore, an elevated concentration in the environment during or following a CSO event would be expected. Licit stimulants including caffeine, nicotine and cotinine are all proposed as markers of untreated wastewater (Buerge et al. 2006; Benotti and Brownawell 2007; Munro et al. 2019; Ramage et al. 2019; Poopipattana et al. 2021). Literature data demonstrates that these compounds are typically present in untreated wastewaters at > 1000 ng L^−1^ with > 90% removal during wastewater treatment removal (Table 3). Buerge et al. (2006) used caffeine and a mass balance approach to estimate that up to 10% of wastewater discharged to the catchment of Lake Greifensee, Switzerland, was untreated. Similarly, paracetamol has been identified as a suitable marker of untreated wastewater discharge due to its high removal during wastewater treatment (Benotti and Brownawell 2007; Ramage et al. 2019). Munro et al. (2019) studied multiple emerging contaminants in a WTP in London, UK, and identified the licit stimulants cocaine and benzoylecgonine as CSO markers. Both compounds had high removal (> 98%) during wastewater treatment as well as low concentration variation in wastewater (Table 3). It should be noted that this may not be the case at other locations due to their recreational use and temporal variability in wastewater concentration (Baker and Kasprzyk-Hordern 2013).

**Table 3:** Emerging contaminants proposed as markers of untreated wastewater

Emerging contaminant	Family/use	Reasoning	Evidence in the environment	Ref.	Literature wastewater data				
					Untreated wastewater concentration (ng L^−1^)	Treatment removal (%)	Untreated wastewater EF	Treated wastewater EF	Ref.
Caffeine	Stimulant	High removal during wastewater treatment and low concentration variation High removal during wastewater treatment High removal during wastewater treatment High removal during wastewater treatment	Elevated concentrations following CSO events	A	1045–150,414	> 95	NA	NA	H
					3400–6600	> 99	NA	NA	I
				B	7000–73,000	81 to > 99	NA	NA	J
			Caffeine loads in stream correlated with rainfall	C	42,400–43,800	> 99	NA	NA	K
					5250–9310	> 99	NA	NA	L
				D	42,000	64	NA	NA	E
			Elevated concentration at site of untreated wastewater discharge Elevated concentrations following CSO events		5094–1,214,375	> 99	NA	NA	M
					74,813	92	NA	NA	N
					135,883–184,819	-	NA	NA	O
Theophylline	Bronchodilator	High removal during wastewater treatment	Elevated concentrations following CSO events	D	1745–107,915	> 93	NA	NA	H
					146,500	95	NA	NA	N
					16,765–237,345	99	NA	NA	M
					75,413–137,196	-	NA	NA	O
Nicotine	Stimulant	High removal during wastewater treatment	No decrease in environmental concentration following CSO event.	E	17,000	87	NA	NA	E
					87–9086	> 95	NA	NA	H
					50–89,600	65 to 99	NA	NA	P
					4874–11,866	99	NA	NA	M
					7750	98	NA	NA	N
					3340–8562	-	NA	NA	O
Cotinine	Stimulant metabolite	High removal during wastewater treatment	Elevated concentration at site of untreated wastewater discharge	C	1972	82	NA	NA	N
					1882–2437	-	NA	NA	O
					44–66	> 69	NA	NA	H
					780–2880	91 to 99	NA	NA	J
					3333–4892	> 99	NA	NA	M
Paracetamol	Analgesic	High removal during wastewater treatment High removal during wastewater treatment High removal during wastewater treatment	No decrease in concentration following CSO event. Elevated concentration at site of untreated wastewater discharge Elevated concentrations following CSO events	E	61,000	99	NA	NA	E
					68,107–482,687	≥ 92	NA	NA	Q
				C	7100–11,400	> 99	NA	NA	R
					-	> 90	NA	NA	S
				D	136,887–343,620	> 99	NA	NA	M
					138,164	99	NA	NA	N
Ibuprofen	NSAID	EF change during wastewater treatment.	Surface water EFs were in the range of sewage overflows.	F	-	-	0.73	0.50	F
					9055–15,780	95	-	-	M
					968–6328	> 85	-	-	Q
					990–3300	> 95	0.85–0.89	0.47–0.67	T
					1213–2058	-	0.79–0.86	0.63–0.68	U
					-	17 to 99	0.73–0.90	0.60–0.76	V
					12,907	90	-	-	N
Naproxen	NSAID	EF change during wastewater treatment due to chiral inversion.	Surface water EFs were in the range of sewage overflows.	F	-	-	> 0.96	0.65–0.92	F
					2925–5455	47	-	-	M
					400–3504	> 57	-	-	Q
					30–430	-	> 0.99	0.88–0.91	W
					1067–3202	-	0.98–0.99	0.93–0.96	U
					-	24 to 89	0.88–0.90	0.71–0.86	V
					13,660	74	-	-	N
Cocaine	Stimulant	High removal during wastewater treatment and low concentration variation	Elevated environmental concentrations following CSO events.	A	882–1575	> 99	NA	NA	A
					21–1837	25 to ≥ 99	NA	NA	Q
					5–209	37 to 91	NA	NA	H
					92–753	93 to > 99	NA	NA	X
					195–961	96	NA	NA	Y
					430	83	NA	NA	N
					397–694	-	NA	NA	O
Benzoylecgonine	Stimulant metabolite	High removal during wastewater treatment and low concentration variation	Elevated environmental concentrations following CSO events.	A	1973–2544	> 98	NA	NA	A
					126–3715	No removal to ≥ 99	NA	NA	Q
					16–567	27 to 81	NA	NA	H
					322–2258	93 to 99	NA	NA	X
					545–3790	88	NA	NA	Y
					1247	69	NA	NA	N
					754–1604	-	NA	NA	O
Amphetamine	Stimulant	High removal and change in EF during wastewater treatment.	Elevated concentration at site of untreated wastewater discharge with EF value (0.43) typical of untreated UK wastewater	C	255–12,020	≥ 95	-	-	Q
					64–368	> 99	0.32–0.46	-	Z
					3–3113	≥ 89	0.16–0.44	0.00–0.29	AA
					291–412	-	0.41–0.43	-	O
					17–3113	≥ 89	-	-	H
					288	77	-	-	N
Propranolol	Beta-blocker	Change in EF during wastewater treatment	EF values in surface waters with untreated wastewater discharges were similar to EF values in untreated wastewater.	G	13–250	-	0.49–0.54	0.30–0.44	G
					54–100	28	-	-	A
					110–1962	No removal to 35	-	-	Q
					108–1130	59 to 78	-	-	R
					122	5	-	-	N
					28–56	No removal to 34	0.37–0.46	0.39–0.45	AB

A, Munro et al. 2019; B, Buerge et al. 2003; C, Ramage et al. 2019; D, Poopipattana et al. 2021; E, Benotti and Brownawell 2007; F, Khan et al. 2014; G, Fono and Sedlak 2005; H, Baker and Kasprzyk-Hordern 2013; I, Sui et al. 2010; J, Buerge et al. 2006; K, Thomas and Foster 2005; L, Froehner et al. 2011; M, Archer et al. 2017; N, Petrie et al. 2016; O, Castrignanò et al. 2016; P, Ekpeghere et al. 2018; Q, Kasprzyk-Hordern et al. 2009; R, Radjenović et al. 2009; S, Matsuo et al. 2011; T, Buser et al. 1999; U, Caballo et al., 2015; V, Matamoros et al. 2009; W, Suzuki et al. 2014; X, van Nuijs et al. 2009; Y, Postigo et al. 2010; Z, Kasprzyk-Hordern et al. 2010; AA, Kasprzyk-Hordern and Baker 2012; AB, López-Serna et al. 2013

Key: NSAID, nonsteroidal anti-inflammatory; EF, enantiomeric fraction; NA, not applicable; -, not measured; CSO, combined sewer overflow; SO, sewage overflow

Other than removal efficiency, several studies report the change in enantiomeric composition of chiral emerging contaminants during wastewater treatment as a means of distinguishing between treated and untreated wastewater discharges (Fono and Sedlak 2005; Khan et al. 2014; Ramage et al. 2019). Chiral compounds have one or more stereogenic centre in their structure. A stereogenic centre is typically an atom with all bonded substituents being different. Enantiomers of a chiral compound have different spatial arrangement of atoms around the stereogenic centre but the same chemical structure (Sanganyado et al. 2017). Therefore, enantiomers have identical physicochemical properties, but due to difference in their three-dimensional shape, behave differently in chiral environments. This results in enantiospecific differences in their metabolism within the body and behaviour during wastewater treatment (Kasprzyk-Hordern 2010). Typically, enantiomeric fraction (EF) is used to describe their enantiomeric composition:

1 $$ EF=\frac{\left(+\right)}{\left[\left(+\right)+\left(-\right)\right]} $$

Here *(+)* is the concentration of the (+)*-*enantiomer and *(-)* is the concentration of the (−)*-*enantiomer. Enantiomers are assigned (+) or (−) depending on the direction they rotate polarised light ((+) is in clockwise direction and (−) is in counterclockwise direction). Therefore, using this approach, an EF value of 0.5 represents a racemic composition (equal concentration of both enantiomers), whereas an EF vale of 0.0 or 1.0 signifies the presence of one enantiomer only. Approximately half of all drugs are chiral (Kasprzyk-Hordern 2010); therefore, determining their enantiomeric composition in wastewater matrices (as well as receiving waters) can then be used to identify CSO discharges in the environment.

Fono and Sedlak (2005) found that the enantiomeric composition of the beta-blocking drug propranolol could be used to identify untreated wastewater discharges (Table 3). The EF value of propranolol in untreated wastewater from five different WTPs in California and New York, USA, was 0.50 ± 0.02. Following biological wastewater treatment, the EF value was reduced to ≤ 0.42 (Fono and Sedlak 2005). The change in EF value of propranolol during wastewater treatment is considered a result of enantioselective degradation (Ribeiro et al. 2013), whereby one enantiomer is degraded at a faster rate than the other. In surface waters with known or suspected untreated wastewater discharges the EF value was ~ 0.50, whereas surface waters with predominantly treated effluent discharges had EF values similar to effluent (Fono and Sedlak 2005).

Similarly, Khan et al. (2014) proposed the use of the NSAIDs naproxen and ibuprofen as markers of untreated wastewater discharges. Both drugs were subject to considerable changes in enantiomeric composition during biological wastewater treatment (Table 3). The EF value of naproxen and ibuprofen in wastewater overflows was > 0.96 and 0.73, respectively. In treated effluents, the EF value of naproxen was reduced to 0.65–0.92 and ibuprofen to 0.50 (Khan et al. 2014). Naproxen is dispensed in medications as the single enantiomer form *S(+)-*naproxen. However, both naproxen and ibuprofen are unlike most other pharmaceuticals in that they can undergo chiral inversion whereby one enantiomer can convert into its antipode (Wsól et al. 2004). This explains the presence of *R(-)-*naproxen in treated effluent and the considerable change to EF values observed.

Ramage et al. (2019) found the stimulant amphetamine to be present in surface water at a site of suspected untreated wastewater discharge in North-East Scotland, UK. The EF value was 0.43 which is typical for untreated wastewaters in the UK (Castrignanò et al. 2016, 2018). Amphetamine is considered readily degradable with degradation favouring *S(+)-*amphetamine (Bagnall et al. 2013). In treated effluents, amphetamine is often not detected, or where it is present, the EF value is < 0.30 due to enantioselective degradation (Table 3). Many other drugs are subject to considerable enantioselective changes during wastewater treatment such as 3,4-methylenedioxymethamphetamine (Kasprzyk-Hordern and Baker 2012; Evans et al. 2016), atenolol (Kasprzyk-Hordern and Baker 2012) and fluoxetine (Andrés-Costa et al. 2017) which could also make them suitable markers of CSO discharges. Understanding the enantiospecific behaviour of chiral analytes in the environment also needs considered as changes can occur here (Bagnall et al. 2013).

**Fig. 2:**
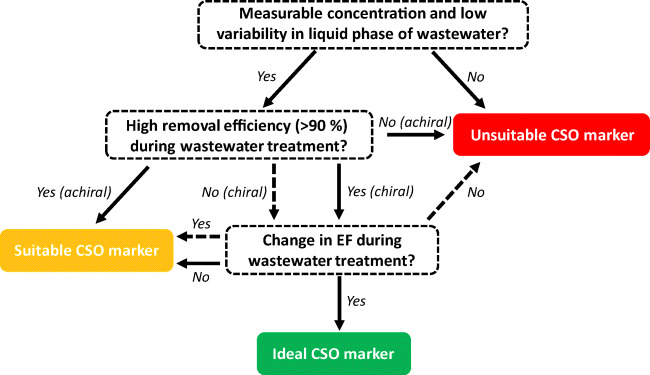
Proposed framework for the selection of emerging contaminants as markers of combined sewer overflows. Key: EF, enantiomeric fraction; CSO, combined sewer overflow

Outlined is a proposed framework to help identify chiral and achiral emerging contaminants that may be suitable markers of CSO discharge (Fig. 2). The use of this framework requires care and site-specific data needs used. For example, the enantiomeric behaviour of drugs can vary between locations (and WTPs). López-Serna et al. (2013) found little change in EF of propranolol during wastewater treatment which is different to the observations of Fono and Sedlak (2005) (Table 3). Emerging contaminant removal during wastewater treatment can also vary between WTPs. To demonstrate, Kasprzyk-Hordern et al. (2009) reported removals of cocaine and benzoylecgonine ranging from little or no removal to ≥ 98% (Table 3).

## Effect of combined sewer overflows to emerging contaminants concentrations in the environment

CSO discharges can influence emerging contaminant concentrations in the environment considering the differences observed for some compounds between CSO and effluent discharges (Benotti and Brownawell 2007; Munro et al. 2019). This can have biological significance for exposed organisms. CSO events could lead to acute exposure to elevated emerging contaminant concentrations. Alternatively, they can maintain dry weather concentrations relevant for chronic exposure, whereby increased dilution of WTP effluent is compensated for by CSO discharges (Benotti and Brownawell 2007). Several studies have attempted to investigate the effect of CSOs on environmental concentrations of wastewater derived emerging contaminants (Table 2). These multi-compound studies use grab sampling to capture changes to emerging contaminant concentrations. Sampling frequency varies from daily (Munro et al. 2019) to monthly (Kay et al. 2017), or targeted following CSO events or rainfall which are then then compared to dry weather samples (Benotti and Brownawell 2007; Ryu et al. 2014; Launay et al. 2016).

**Fig. 3:**
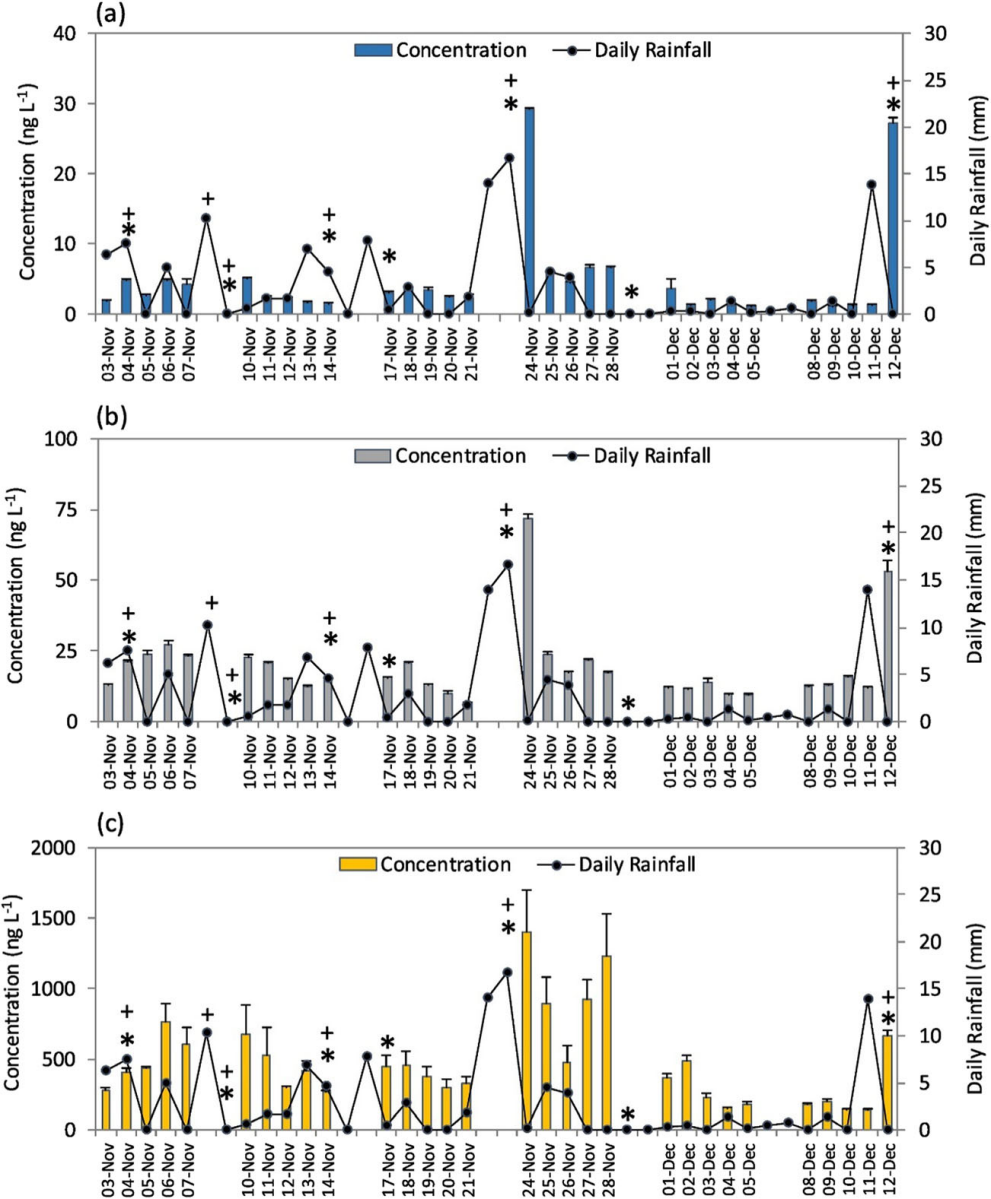
Occurrence of cocaine, benzoylecgonine and caffeine in the River Thames during November–December 2014. Bars represent mean concentration from two replicates and whiskers represent the maximum concentration measured. Key: +, storm water and untreated wastewater were combined and released directly into river; *, storm water and treated wastewater were combined and released into environment (reproduced from Munro et al. 2019)

Munro et al. (2019) studied 31 emerging contaminants at two locations in the tidal region of the River Thames. Daily samples were collected during workdays for six weeks (Table 2). During this time, six CSO events took place which discharged untreated wastewaters and storm waters. Elevated concentrations of cocaine, benzoylecgonine and caffeine were noted on two occasions (see 24th Nov and 12th Dec; Fig. 3). These concentrations were within an order of magnitude of those concentrations determined when CSO events did not take place (Munro et al. 2019). There are several reasons why elevated concentrations were not observed following other CSO events. This could be due to the scale of the CSO discharge or the discharge event/sample collection occurring at the top of the tidal phase. Both of which can result in adequate dilution such that there is no significant effect on concentration. Poopipattana et al. (2021) sampled the Tokyo Estuary, Japan, for five emerging contaminants (caffeine, theophylline, paracetamol, carbamazepine and crotamiton) following heavy rainfall. Increased concentrations in the estuary, compared with dry weather conditions, were observed for caffeine, theophylline and paracetamol, attributed to their high removal during wastewater treatment.

Benotti and Brownawell (2007) investigated the effect of CSO discharges to 21 emerging contaminants in Jamaica Bay Estuary, USA. Samples were collected from 24 different locations, albeit at lower frequency, with sampling focused at times following CSO events. The concentration for most compounds reduced following CSO discharges due to greater dilution in the estuary caused by rainfall. However, concentrations of nicotine and paracetamol either maintained their dry weather concentration or increased in concentration following CSO discharges (Benotti and Brownawell, 2007). Nevertheless, assessing the effect of CSO discharges to emerging contaminant concentrations in estuarine waters is challenging as the tidal phase adds further complexity to the interpretation of data.

Several studies have investigated the possible effects of CSO discharges to emerging contaminants in rivers upstream of tidal waters (Ryu et al. 2014; Launay et al. 2016; Kay et al. 2017). Kay et al. (2017) investigated five emerging contaminants in the Aire and Calder catchments, UK, over 18 months. Samples were collected monthly and although variability in concentration was observed, no correlation to rainfall or CSO discharges was reported. Ryu et al. (2014) assessed the influence of a CSO discharge event to emerging contaminant concentrations in the Jung-rang Creek, South Korea (Table 2). A total of 29 compounds were studied with cumulative concentrations (sum of all compounds) in surface water reducing by 34% following the CSO event (Ryu et al. 2014). However, it is difficult to draw conclusions as only a single grab sample per sampling site was collected during dry weather conditions, and one under wet weather conditions following the CSO event.

Launay et al. (2016) investigated the effect of CSO discharges to emerging contaminant concentrations in the Körsch catchment, Germany. Surface waters were sampled during dry weather conditions to obtain baseline information and following wet weather to assess the impact of CSO discharges. Specifically, diclofenac concentrations were compared to its proposed annual average environmental quality standard (AA-EQS) of 100 ng L^−1^ (European Commission 2012). The AA-EQS was exceeded downstream of the CSO discharge (which is upstream of the WTP effluent discharge) during wet weather in 25% of samples (*n* = 4) (Table 2). Although this only represents a single sample (concentration = 280 ng L^−1^), the AA-EQS was not exceeded here during dry weather (*n* = 9). Diclofenac concentrations downstream of the WTP effluent discharge were lower during wet weather. Nevertheless, all samples downstream of the WTP effluent discharge under both dry and wet weather conditions exceeded the AA-EQS (Launay et al. 2016).

## Mitigation and treatment strategies for emerging contaminants in combined sewer overflows

Mitigation and treatment strategies can be implemented to reduce the contribution of CSOs to emerging contaminants found in the environment. This is particularly important where the contribution of CSOs alone can exceed emerging contaminant toxicity thresholds. The replacement of existing combined sewers with separate sewer systems can improve river water quality (e.g., see Fig. 1) but is often cost prohibitive and difficult to achieve in high-density locations. Ideally, other mitigation strategies can be adopted which avoid the need for end-of-pipe technologies. External storage of stormwater prior to entering combined sewers during high flows can reduce CSOs. However, available space at suitable locations within the catchment can be limited. Sustainable urban drainage systems (SuDS) can also be used to help reduce the volume of stormwater entering combined sewers. SuDS techniques include bioretention cells, permeable pavements, rain barrels and green roofs (Joshi et al. 2021). Joshi et al. (2021) modelled the impact of these four SuDs techniques to CSOs in the Fehraltorf catchment, Switzerland. This is a moderately sized catchment in which 82 ha is connected to a combined sewer and the WTP has a treatment capacity of 180 L s^-1^. Findings revealed that such approaches could reduce CSO volumes in this catchment by 50 to 99 % under a range of scenarios (Joshi et al. 2021).

A considerable reduction in CSOs can be achieved by real-time monitoring of sewer flows and making use of existing pipeline capacity. For example, Carbone et al. (2014) proposed the use of ‘smart’ gates that adjust themselves during storm events to optimise upstream sewer capacity. Several researchers have developed predictive models to minimise CSO discharges (Zhao et al. 2019; Snodgrass et al. 2018; Rathnayake and Faisal Anwar 2019). Troutman et al. (2020) proposed a load-balancing algorithm to control storage assets within the catchment to improve flow dynamics at the WTP. This is facilitated with the use of wireless technologies and autonomous control of gates, valves and pumps within the sewer network.

Alternatively, treatment strategies are adopted to treat CSOs before they enter the environment. These can be nature-based solutions such as constructed wetlands which can remove emerging contaminants from CSOs (Scheurer et al. 2015; Tondera et al. 2019). Furthermore, these systems can provide flood mitigation by reducing the intensity of peak flows (Rizzo et al. 2018). Vertical flow constructed wetlands also known as retention soil filters are popular and consist of a planted media bed which water percolates. Soil was initially used as the bed media, but sand is now popular with up to 20% calcium carbonate (broken limestone) to stabilise pH (Tondera et al. 2019). Such systems are often planted with the common reed (*Phragmites australis*) (Scheurer et al. 2015; Brunsch et al. 2018, 2020). Biological degradation and sorption of emerging contaminants can take place in the media bed (Petrie et al. 2018). The presence of plants is known to increase emerging contaminant removal in constructed wetlands (Matamoros et al. 2012; Hijosa-Valsero et al. 2016). Plant roots act as a surface for biofilm growth; they pump and release oxygen, insulate against low temperatures and improve retention of solid particles (Tanner 2001; Kyambadde et al. 2004). Plants can also take up and metabolise emerging contaminants (He et al. 2017; Petrie et al. 2018).

Scheurer et al. (2015) investigated the removal of several emerging contaminants in a full-scale retention soil filter treating CSO discharges. The soil bed was planted with *P. australis* and received intermittent CSO discharges (average of 40–60 events per year). Removal efficiencies were greatest for those readily biodegradable contaminants paracetamol (98%) and ibuprofen (94%) (Scheurer et al. 2015). The removal observed for most compounds were comparable to removal in the activated sludge WTP which treats the entire wastewater flow during dry weather. However, several compounds were removed to a greater extent by the retention soil filter. In particular, the average removal of diclofenac was 87% whereas only 14% was removed by activated sludge treatment (Scheurer et al. 2015). However, longer term studies indicate a loss of capacity to remove emerging contaminants. For example, Tondera et al. (2019) reported diclofenac removals of 67 % in a retention soil filter which was operated for seven years. This reduced to 34% after 10 years of operation which the authors suggest a replacement of bed media may be required once performance begins to reduce (Tondera et al. 2019). A temperature dependency has also been observed whereby removal efficiencies of bisphenol-A during winter were 53% and during summer were 90% (Tondera et al. 2019). Pilot-scale studies revealed that the dry period between CSO events did not have an impact to removal efficiency of bisphenol-A, carbamazepine, diclofenac, metoprolol, and sulfamethoxazole (Ruppelt et al. 2020). However, it should be noted that no removal of carbamazepine and sulfamethoxazole was observed by retention soil filters. The recalcitrance of carbamazepine in the environment and during biological wastewater treatment is well established (Zhang et al. 2008).

It is also possible to make use of CSO treatment methods to polish WTP effluents during dry weather (acting as a tertiary treatment for WTP effluent). Such an approach makes continual use of WTP infrastructure and reduces the release of emerging contaminants from two emission pathways. Brunsch et al. (2020) utilised a pilot-scale soil retention filter to assess the effectiveness of such an approach. Removals of several emerging contaminants were > 50% from CSOs and WTP effluent, with caffeine and metformin reaching > 99% from CSOs (Brunsch et al. 2020). It was noted that following CSO treatment compounds sorbed to filter material or present in pore water within the soil retention filter were washed out by WTP effluent. However, this could be counteracted by an 18 h dry period between treating CSOs and WTP effluents (Brunsch et al. 2020).

A further option for the treatment of emerging contaminants in CSOs is by technological compact treatments (Botturi et al. 2020). For example, Botturi et al. (2020) describe the installation of a pilot scale modular system adopting rotating belt filtration, granular activated carbon (GAC) filtration and UV disinfection. Although such processes have been studied for the removal of emerging contaminants previously (e.g. see Grover et al. 2011; Yang et al. 2014), studies on their application for emerging contaminants in CSOs is currently lacking. Both high water volumes for treatment and high solids loading are likely to influence their feasibility. Advanced treatment processes are often applied to treated effluents which have low solids loading. Therefore, the removal of suspended particulates prior to adsorption or chemical treatment of CSOs is needed. The belt filtration processes utilised by Botturi et al. (2020) sieves CSOs through a 350 μm mesh prior to GAC and UV treatment. In another study, Jung et al. (2015) investigated the adsorption of naproxen and paracetamol to activated biochar under synthetically prepared CSO wastewater. The removals achieved for naproxen and paracetamol were 98% and 94% (Jung et al. 2015). Although these results are promising, further work is needed to establish the use and feasibility of such treatment at a suitable scale for CSOs.

## Conclusion

Investigating the contribution of CSOs to wastewater-derived emerging contaminants found in the environment poses several challenges. This review demonstrates that significant progress has been made in this research area. Nevertheless, further research is now needed to further our understanding of CSOs and their impact. Due to the impact of CSOs on the environment being catchment-specific, studies on emerging contaminants are needed under a range of conditions. This demands a more systematic monitoring strategy from selection of the sampling locations to the choice of analytes to measure. Monitoring CSOs themselves is needed to apportion their contribution to released loads of emerging contaminants to the environment. The selection of effective emerging contaminant markers of CSO discharges is essential. Findings from such studies will then enable appropriate decision making at catchment level on the need for any mitigation and treatment strategies.

## Data Availability

The datasets used and/or analysed during the current study are available from the corresponding author on reasonable request.

## References

[CR1] Abdellatif M, Atherton W, Alkhaddar RM, Osman YZ (2015). Quantitative assessment of sewer overflow performance with climate change in northwest England. Hydrol Sci J.

[CR2] Andrés-Costa MJ, Proctor K, Sabatini MT, Gee AP, Lewis SE, Pico Y, Kasprzyk-Hordern B (2017). Enantioselective transformation of fluoxetine in water and its ecotoxicological relevance. Sci Rep.

[CR3] Archer E, Petrie B, Kasprzyk-Hordern B, Wolfaardt GM (2017). The fate of pharmaceuticals and personal care products (PPCPs), endocrine disrupting contaminants (EDCs), metabolites and illicit drugs in a WWTW and environmental waters. Chemosphere.

[CR4] Bagnall J, Malia L, Lubben A, Kasprzyk-Hordern B (2013). Stereoselective biodegradation of amphetamine and methamphetamine in river microcosms. Water Res.

[CR5] Baker DR, Kasprzyk-Hordern B (2013). Spatial and temporal occurrence of pharmaceuticals and illicit drugs in the aqueous environment and during wastewater treatment: New developments. Sci Total Environ.

[CR6] Benotti MJ, Brownawell BJ (2007). Distributions of pharmaceuticals in an urban estuary during both dry- and wet-weather conditions. Environ Sci Technol.

[CR7] Botturi A, Ozbayram EG, Tondera K, Gilbert NI, Rouault P, Caradot N, Gutierrez O, Daneshgar S, Frison N, Akyol Ç, Foglia A, Eusebi AL, Fatone F (2020) Combined sewer overflows: A critical review on best practice and innovative solutions to mitigate impacts on environment and human health. Crit Rev Environ Sci Technol:1–34. 10.1080/10643389.2020.1757957

[CR8] Brodin T, Fick J, Jonsson M, Klaminder J (2013). Dilute concentrations of a psychiatric drug alter behavior of fish from natural populations. Science.

[CR9] Brunsch AF, Zubieta Florez P, Langenhoff AAM, ter Laak TL, Rijnaarts HHM (2020). Retention soil filters for the treatment of sewage treatment plant effluent and combined sewer overflow. Sci Total Environ.

[CR10] Brunsch AF, ter Laak TL, Christoffels E, Rijnaarts HHM, Langenhoff AAM (2018). Retention soil filter as post-treatment step to remove micropollutants from sewage treatment plant effluent. Sci Total Environ.

[CR11] Buerge IJ, Poiger T, Müller MD, Buser HR (2003). Caffeine, an anthropogenic marker for wastewater contamination of surface waters. Environ Sci Technol.

[CR12] Buerge IJ, Poiger T, Müller MD, Buser HR (2006). Combined sewer overflows to surface waters detected by the anthropogenic marker caffeine. Environ Sci Technol.

[CR13] Buser HR, Poiger T, Muller MD (1999). Occurrence and environmental behavior of the chiral pharmaceutical drug ibuprofen in surface waters and in wastewater. Environ Sci Technol.

[CR14] Caballo C, Sicilia MD, Rubio S (2015). Enantioselective determination of representative profens in wastewater by a single-step sample treatment and chiral liquid chromatography-tandem mass spectrometry. Talanta.

[CR15] Carbone M, Garofalo G, Piro P (2014). Decentralized real time control in combined sewer system by using smart objects. Procedia Eng.

[CR16] Carvalho RN, Ceriani L, Ippolito A, Lettieri T (2015) Development of the first Watch List under the Environmental Quality Standards Directive, JRC Technical Report, European Commission.

[CR17] Castrignanò E, Yang Z, Bade R, Baz-Lomba JA, Castiglioni S, Causanilles A, Covaci A, Gracia-Lor E, Hernandez F, Kinyua J, McCall AK, van Nuijs ALN, Ort C, Plósz BG, Ramin P, Rousis NI, Ryu Y, Thomas KV, de Voogt P, Zuccato E, Kasprzyk-Hordern B (2018). Enantiomeric profiling of chiral illicit drugs in a pan-European study. Water Res.

[CR18] Castrignanò E, Lubben A, Kasprzyk-Hordern B (2016). Enantiomeric profiling of chiral drug biomarkers in wastewater with the usage of chiral liquid chromatography coupled with tandem mass spectrometry. J Chromatogr A.

[CR19] Chèvre N, Coutu S, Margot J, Wynn HK, Bader HP, Scheidegger R, Rossi L (2013). Substance flow analysis as a tool for mitigating the impact of pharmaceuticals on the aquatic system. Water Res.

[CR20] Cleuvers M (2004). Mixture toxicity of the anti-inflammatory drugs diclofenac, ibuprofen, naproxen, and acetylsalicylic acid. Ecotoxicol Environ Saf.

[CR21] Cortes LG, Marinov D, Sanseverino I, Cuenca AN, Niegowska M, Rodriguez EP, Lettieri T (2020) Selection of substances for the 3rd Watch List under the Water Framework Directive, EUR 30297 EN, Luxembourg: Publications Office of the European Union, ISBN 978-92-76-19426-2, 10.2760/194067,JRC121346.

[CR22] DEFRA (2015) Creating a River Thames fit for our future - An updated strategic and economic case for the Thames Tideway Tunnel (publishing.service.gov.uk)

[CR23] Del Río H, Suárez J, Puertas J, Ures P (2013). PPCPs wet weather mobilization in a combined sewer in NW Spain. Sci Total Environ.

[CR24] Ekpeghere KI, Sim WJ, Lee HJ, Oh JE (2018). Occurrence and distribution of carbamazepine, nicotine, estrogenic compounds, and their transformation products in wastewater from various treatment plants and the aquatic environment. Sci Total Environ.

[CR25] Ellis JB (2006). Pharmaceutical and personal care products (PPCPs) in urban receiving waters. Environ Pollut.

[CR26] Environment Agency (2020) Consented Discharges to Controlled Waters with Conditions (data.gov.uk)

[CR27] European Commission (2012) European Commission Proposal for a Directive of the European Parliament and of the Council Amending Directives 2000/60/EC and 2008/105/EC as Regards Priority Substances in the Field of Water Policy.

[CR28] Evans SE, Bagnall J, Kasprzyk-Hordern B (2016). Enantioselective degradation of amphetamine-like environmental micropollutants (amphetamine, methamphetamine, MDMA and MDA) in urban water. Environ Pollut.

[CR29] Fono LJ, Sedlak DL (2005). Use of the chiral pharmaceutical propranolol to identify sewage discharges into surface waters. Environ Sci Technol.

[CR30] Froehner S, Piccioni W, Machado KS, Aisse MM (2011). Removal capacity of caffeine, hormones, and bisphenol by aerobic and anaerobic sewage treatment. Water Air Soil Pollut.

[CR31] Gasperi J, Zgheib S, Cladière M, Rocher V, Moilleron R, Chebbo G (2012). Priority pollutants in urban stormwater: Part 2 - Case of combined sewers. Water Res.

[CR32] Gros M, Petrović M, Barceló D (2007). Wastewater treatment plants as a pathway for aquatic contamination by pharmaceuticals in the ebro river basin (northeast Spain). Environ Toxicol Chem.

[CR33] Grover DP, Zhou JL, Frickers PE, Readman JW (2011). Improved removal of estrogenic and pharmaceutical compounds in sewage effluent by full scale granular activated carbon: Impact on receiving river water. J Hazard Mater.

[CR34] Hajj-Mohamad M, Darwano H, Duy SV, Sauvé S, Prévost M, Arp HPH, Dorner S (2017). The distribution dynamics and desorption behaviour of mobile pharmaceuticals and caffeine to combined sewer sediments. Water Res.

[CR35] He Y, Langenhoff AAM, Sutton NB, Rijnaarts HHM, Blokland MH, Chen F, Huber C, Schröder P (2017). Metabolism of Ibuprofen by Phragmites australis: Uptake and Phytodegradation. Environ Sci Technol.

[CR36] Hijosa-Valsero M, Reyes-Contreras C, Domínguez C, Bécares E, Bayona JM (2016). Behaviour of pharmaceuticals and personal care products in constructed wetland compartments: Influent, effluent, pore water, substrate and plant roots. Chemosphere.

[CR37] Hughes SR, Kay P, Brown LE (2013). Global synthesis and critical evaluation of pharmaceutical data sets collected from river systems. Environ Sci Technol.

[CR38] Joshi P, Leitão JP, Maurer M, Bach PM (2021). Not all SuDS are created equal: Impact of different approaches on combined sewer overflows. Water Res.

[CR39] Jung C, Oh J, Yoon Y (2015). Removal of acetaminophen and naproxen by combined coagulation and adsorption using biochar: influence of combined sewer overflow components. Environ Sci Pollut Res.

[CR40] Kasprzyk-Hordern B (2010). Pharmacologically active compounds in the environment and their chirality. Chem Soc Rev.

[CR41] Kasprzyk-Hordern B, Kondakal VVR, Baker DR (2010). Enantiomeric analysis of drugs of abuse in wastewater by chiral liquid chromatography coupled with tandem mass spectrometry. J Chromatogr A.

[CR42] Kasprzyk-Hordern B, Baker DR (2012). Enantiomeric profiling of chiral drugs in wastewater and receiving waters. Environ Sci Technol.

[CR43] Kasprzyk-Hordern B, Dinsdale RM, Guwy AJ (2009). The removal of pharmaceuticals, personal care products, endocrine disruptors and illicit drugs during wastewater treatment and its impact on the quality of receiving waters. Water Res.

[CR44] Kay P, Hughes SR, Ault JR, Ashcroft AE, Brown LE (2017). Widespread, routine occurrence of pharmaceuticals in sewage effluent, combined sewer overflows and receiving waters. Environ Pollut.

[CR45] Khan SJ, Wang L, Hashim NH, Mcdonald JA (2014). Distinct enantiomeric signals of ibuprofen and naproxen in treated wastewater and sewer overflow. Chirality.

[CR46] Kidd KA, Blanchfield PJ, Mills KH, Palace VP, Evans RE, Lazorchak JM, Flick RW (2007). Collapse of a fish population after exposure to a synthetic estrogen. Proc Natl Acad Sci USA.

[CR47] Kostich MS, Batt AL, Lazorchak JM (2014). Concentrations of prioritized pharmaceuticals in effluents from 50 large wastewater treatment plants in the US and implications for risk estimation. Environ Pollut.

[CR48] Kyambadde J, Kansiime F, Gumaelius L, Dalhammar G (2004). A comparative study of Cyperus papyrus and Miscanthidium violaceum-based constructed wetlands for wastewater treatment in a tropical climate. Water Res.

[CR49] Lange FT, Scheurer M, Brauch HJ (2012). Artificial sweeteners-A recently recognized class of emerging environmental contaminants: A review. Anal Bioanal Chem.

[CR50] Launay MA, Dittmer U, Steinmetz H (2016). Organic micropollutants discharged by combined sewer overflows – Characterisation of pollutant sources and stormwater-related processes. Water Res.

[CR51] Li WC (2014). Occurrence, sources, and fate of pharmaceuticals in aquatic environment and soil. Environ Pollut.

[CR52] López-Serna R, Kasprzyk-Hordern B, Petrović M, Barceló D (2013). Multi-residue enantiomeric analysis of pharmaceuticals and their active metabolites in the Guadalquivir River basin (South Spain) by chiral liquid chromatography coupled with tandem mass spectrometry. Anal Bioanal Chem.

[CR53] Madoux-Humery AS, Dorner S, Sauvé S, Aboulfadl K, Galarneau M, Servais P, Prévost M (2013). Temporal variability of combined sewer overflow contaminants: Evaluation of wastewater micropollutants as tracers of fecal contamination. Water Res.

[CR54] Matamoros V, Hijosa M, Bayona JM (2009). Assessment of the pharmaceutical active compounds removal in wastewater treatment systems at enantiomeric level. Ibuprofen and naproxen. Chemosphere.

[CR55] Matamoros V, Nguyen LX, Arias CA, Salvadó V, Brix H (2012). Evaluation of aquatic plants for removing polar microcontaminants: A microcosm experiment. Chemosphere.

[CR56] Matsuo H, Sakamoto H, Arizono K, Shinohara R (2011). Behavior of pharmaceuticals in waste water treatment plant in Japan. B Environ Contam Toxicol.

[CR57] Munro K, Martins CPB, Loewenthal M, Comber S, Cowan DA, Pereira L, Barron LP (2019). Evaluation of combined sewer overflow impacts on short-term pharmaceutical and illicit drug occurrence in a heavily urbanised tidal river catchment (London, UK). Sci Total Environ.

[CR58] Mutzner L, Bohren C, Mangold S, Bloem S, Ort C (2020). Spatial differences among micropollutants in sewer overflows: a multisite analysis using passive samplers. Environ Sci Technol.

[CR59] Mutzner L, Vermeirssen ELM, Mangold S, Maurer M, Scheidegger A, Singer H, Booij K, Ort C (2019). Passive samplers to quantify micropollutants in sewer overflows: accumulation behaviour and field validation for short pollution events. Water Res.

[CR60] Ort C, Lawrence MG, Reungoat J, Mueller JF (2010). Sampling for PPCPs in wastewater systems: Comparison of different sampling modes and optimization strategies. Environ Sci Technol.

[CR61] Petrie B, Barden R, Kasprzyk-Hordern B (2015). A review on emerging contaminants in wastewaters and the environment: Current knowledge, understudied areas and recommendations for future monitoring. Water Res.

[CR62] Petrie B, Rood S, Smith BD, Proctor K, Youdan J, Barden R, Kasprzyk-Hordern B (2018). Biotic phase micropollutant distribution in horizontal sub-surface flow constructed wetlands. Sci Total Environ.

[CR63] Petrie B, Youdan J, Barden R, Kasprzyk-Hordern B (2016). Multi-residue analysis of 90 emerging contaminants in liquid and solid environmental matrices by ultra-high-performance liquid chromatography tandem mass spectrometry. J Chromatogr A.

[CR64] Phillips PJ, Chalmers AT, Gray JL, Kolpin DW, Foreman WT, Wall GR (2012). Combined sewer overflows: An environmental source of hormones and wastewater micropollutants. Environ Sci Technol.

[CR65] Poopipattana C, Suzuki M, Furumai H (2021). Impact of long-duration CSO events under different tidal change conditions on distribution of microbial indicators and PPCPs in Sumida river estuary of Tokyo Bay, Japan. Environ Sci Pollut Res.

[CR66] Postigo C, López de Alda MJ, Barceló D (2010). Drugs of abuse and their metabolites in the Ebro River basin: Occurrence in sewage and surface water, sewage treatment plants removal efficiency, and collective drug usage estimation. Environ Int.

[CR67] Radjenović J, Petrović M, Barceló D (2009). Fate and distribution of pharmaceuticals in wastewater and sewage sludge of the conventional activated sludge (CAS) and advanced membrane bioreactor (MBR) treatment. (2009). Water Res.

[CR68] Ramage S, Camacho-Muñoz D, Petrie B (2019). Enantioselective LC-MS/MS for anthropogenic markers of septic tank discharge. Chemosphere.

[CR69] Rathnayake U, Faisal Anwar AHM (2019). Dynamic control of urban sewer systems to reduce combined sewer overflows and their adverse impacts. J Hydrol.

[CR70] Ribeiro AR, Afonso CM, Castro PML, Tiritan ME (2013). Enantioselective biodegradation of pharmaceuticals, alprenolol and propranolol, by an activated sludge inoculum. Ecotoxicol Environ Saf.

[CR71] Rizzo A, Bresciani R, Masi F, Boano F, Revelli R, Ridolfi L (2018). Flood reduction as an ecosystem service of constructed wetlands for combined sewer overflow. J Hydrol.

[CR72] Rizzo L, Manaia C, Merlin C, Schwartz T, Dagot C, Ploy MC, Michael I, Fatta-Kassinos D (2013). Urban wastewater treatment plants as hotspots for antibiotic resistant bacteria and genes spread into the environment: A review. Sci Total Environ.

[CR73] Ruppelt JP, Pinnekamp J, Tondera K (2020). Elimination of micropollutants in four test-scale constructed wetlands treating combined sewer overflow: Influence of filtration layer height and feeding regime. Water Res.

[CR74] Ryu J, Oh J, Snyder SA, Yoon Y (2014). Determination of micropollutants in combined sewer overflows and their removal in a wastewater treatment plant (Seoul, South Korea). Environ Monit Assess.

[CR75] Sanganyado E, Lu Z, Fu Q, Schlenk D, Gan J (2017). Chiral pharmaceuticals: A review on their environmental occurrence and fate processes. Water Res.

[CR76] Schertzinger G, Zimmermann S, Sures B (2019). Predicted sediment toxicity downstream of combined sewer overflows corresponds with effects measured in two sediment contact bioassays. Environ Pollut.

[CR77] Scheurer M, Heß S, Lüddeke F, Sacher F, Güde H, Löffler H, Gallert C (2015). Removal of micropollutants, facultative pathogenic and antibiotic resistant bacteria in a full-scale retention soil filter receiving combined sewer overflow. Environ Sci Process Impacts.

[CR78] Schnell S, Bols NC, Barata C, Porte C (2009). Single and combined toxicity of pharmaceuticals and personal care products (PPCPs) on the rainbow trout liver cell line RTL-W1. Aquat Toxicol.

[CR79] Scottish Water (2020) A resilient waste water network | Scottish Water (yourwater.scot)

[CR80] Snodgrass WJ, Dewey R, D’Andrea M, Bishop R, Lei J (2018). Forecasting receiving water response to alternative control levels for combined sewer overflows discharging to Toronto’s Inner Harbour. Aquat Ecosyst Health Manag.

[CR81] Sui Q, Huang J, Deng S, Yu G, Fan Q (2010). Occurrence and removal of pharmaceuticals, caffeine and DEET in wastewater treatment plants of Beijing, China. Water Res.

[CR82] Suzuki T, Kosugi Y, Hosaka M, Nishimura T, Nakae D (2014). Occurrence and behavior of the chiral anti-inflammatory drug naproxen in an aquatic environment. Environ Toxicol Chem.

[CR83] Tanner CC (2001). Plants as ecosystem engineers in subsurface-flow treatment wetlands. Water Sci Technol.

[CR84] Thomas PM, Foster GD (2005). Tracking acidic pharmaceuticals, caffeine, and triclosan through the wastewater treatment process. Environ Toxicol Chem.

[CR85] Tondera K, Ruppelt JP, Pinnekamp J, Kistemann T, Schreiber C (2019). Reduction of micropollutants and bacteria in a constructed wetland for combined sewer overflow treatment after 7 and 10 years of operation. Sci Total Environ.

[CR86] Troutman SC, Love NG, Kerkez B (2020). Balancing water quality and flows in combined sewer systems using real-time control. Environ Sci Water Res Technol.

[CR87] van Nuijs ALN, Pecceu B, Theunis L, Dubois N, Charlier C, Jorens PG, Bervoets L, Blust R, Neels H, Covaci A (2009). Spatial and temporal variations in the occurrence of cocaine and benzoylecgonine in waste- and surface water from Belgium and removal during wastewater treatment. Water Res.

[CR88] Weyrauch P, Matzinger A, Pawlowsky-Reusing E, Plume S, von Seggern D, Heinzmann B, Schroeder K, Rouault P (2010). Contribution of combined sewer overflows to trace contaminant loads in urban streams. Water Res.

[CR89] Wsól V, Skálová L, Szotáková B (2004). Chiral inversion of drugs: Coincidence or principle?. Curr Drug Metab.

[CR90] Yang W, Zhou H, Cicek N (2014). Treatment of organic micropollutants in water and wastewater by UV-based processes: A literature review. Crit Rev Environ Sci Technol.

[CR91] Zhang Y, Geißen SU, Gal C (2008). Carbamazepine and diclofenac: removal in wastewater treatment plants and occurrence in water bodies. Chemosphere.

[CR92] Zhao W, Beach TH, Rezgui Y (2019). Automated model construction for combined sewer overflow prediction based on efficient LASSO Algorithm. IEEE Trans Syst Man Cybern Syst.

[CR93] Zhao X, Zheng Y, Hu S, Qiu W, Jiang J, Gao C, Xiong J, Lu H, Quan F (2021). Improving urban drainage systems to mitigate PPCPs pollution in surface water: a watershed perspective. J Hazard Mater.

[CR94] Zuccato E, Castiglioni S, Bagnati R, Chiabrando C, Grassi P, Fanelli R (2008). Illicit drugs, a novel group of environmental contaminants. Water Res.

